# Analysis of the 3’ untranslated regions of α-tubulin and S-crystallin mRNA and the identification of CPEB in dark- and light-adapted octopus retinas

**Published:** 2008-08-04

**Authors:** Shannan Kelly, Hideki Yamamoto, Laura J. Robles

**Affiliations:** Department of Biology, California State University, Dominguez Hills, Carson, CA

## Abstract

**Purpose:**

We previously reported the differential expression and translation of mRNA and protein in dark- and light-adapted octopus retinas, which may result from cytoplasmic polyadenylation element (CPE)–dependent mRNA masking and unmasking. Here we investigate the presence of CPEs in α-tubulin and S-crystallin mRNA and report the identification of cytoplasmic polyadenylation element binding protein (CPEB) in light- and dark-adapted octopus retinas.

**Methods:**

3’-RACE and sequencing were used to isolate and analyze the 3’-UTRs of α-tubulin and S-crystallin mRNA. Total retinal protein isolated from light- and dark-adapted octopus retinas was subjected to western blot analysis followed by CPEB antibody detection, PEP-171 inhibition of CPEB, and dephosphorylation of CPEB.

**Results:**

The following CPE-like sequence was detected in the 3’-UTR of isolated long S-crystallin mRNA variants: UUUAACA. No CPE or CPE-like sequences were detected in the 3’-UTRs of α-tubulin mRNA or of the short S-crystallin mRNA variants. Western blot analysis detected CPEB as two putative bands migrating between 60-80 kDa, while a third band migrated below 30 kDa in dark- and light-adapted retinas.

**Conclusions:**

The detection of CPEB and the identification of the putative CPE-like sequences in the S-crystallin 3’-UTR suggest that CPEB may be involved in the activation of masked S-crystallin mRNA, but not in the regulation of α-tubulin mRNA, resulting in increased S-crystallin protein synthesis in dark-adapted octopus retinas.

## Introduction

The actin cytoskeleton of octopus photoreceptors undergoes dramatic reorganization in the dark and light: the number of rhabdomeric microvilli increase in the dark, resulting in the growth of rhabdomeres; whereas, there is a decrease in the number of rhabdomeric microvilli in the light, resulting in the diminution of the rhabdomeres [1]. Corresponding with these distinct morphological changes are drastic changes in protein distribution as well as differences in overall mRNA and protein concentration in dark-and light-adapted retinas [2,3]. We have previously reported the differential expression of S-crystallin, which we have shown to bind F-actin, and α-tubulin in light- and dark-adapted octopus retinas. Real-time PCR analysis of light-adapted retinal mRNA showed 1.4 and 2 fold inductions of α-tubulin (unpublished) and S-crystallin, respectively, when compared to dark-adapted retinas [4]. Percent volume analysis of dark-adapted retinal protein showed that α-tubulin (unpublished) and S-crystallin were 2 fold more abundant when compared to light-adapted retinas [4].

In addition to increases in the expression of certain crystallins in light-damaged retinas and in age-related macular degenerative patients, decreases in αA-crystallins expression in the retinas of dystrophic rats have also been shown [5-7]. However, there are no established studies demonstrating the mechanisms by which light or dark adaptation contributes to the differential expression α-tubulin and S-crystallin in the retina. Not only is there structural similarity between the complex camera eyes and photoreceptors of cephalopods and vertebrates, but there is also similarity in some of their crystallin and tubulin expression and function [8-10]. We believe that light and dark exposure may regulate the differential expression of these cytoskeletal genes in octopus retinas, which directly leads to remodeling of the actin- and tubulin-based cytoskeleton. Understanding how light and dark exposure may regulate the differential expression of crystallin and tubulin genes in octopus retinas will facilitate our understanding of cytoskeletal organization in vertebrate photoreceptors.

**Figure 1:**
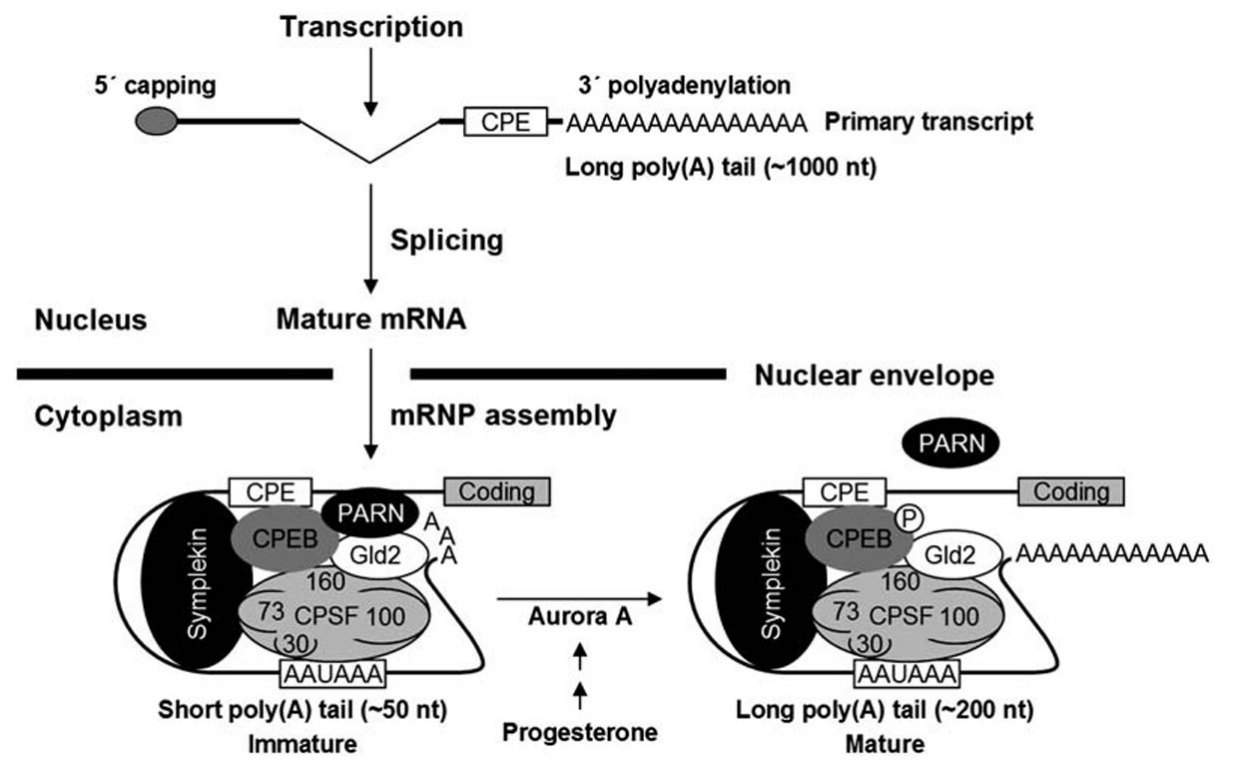
Model of Poly(A) tail dynamics of CPE-containing mRNA. CPEB is always bound to the CPE consensus sequence, indicating CPEB and CPE in both translational repression and translational activation. Prior to exit from the nucleus, mRNAs have long poly(A)^+^ tails that are shortened upon entrance into the cytoplasm by the interaction of CPEB, the poly(A)-specific RNase (PARN), and the poly(A) polymerase, Gld2, thereby promoting translational repression. CPEB, PARN, and Gld2 are held together in the ribonucleoprotein (RNP) complex maintained by the scaffold protein symplekin, which also interacts with the cleavage and polyadenylation specificity factor (CPSF). During translational activation CPEB is phosphorylated by Aurora A, resulting in the release of PARN from CPEB and Gld2 and subsequent initiation of poly(A)^+^ tail elongation by Gld2. Reprinted from reference [27], *Molecular Cell*, 24, Kim Jong Heon, Richter Joel D, *Opposing polymerase-deadenylase activities regulate cytoplasmic polyadenylation*, pages 173-183, copyright 2006, with permission from Elsevier Limited, Oxford, UK.

One of many common effectors of differential gene expression is post-transcriptional regulation, which is known to occur through translational repression by micro RNAs (miRNAs) and short interfering RNAs (siRNAs). Post-transcriptional regulation is also known to occur through mRNA degradation, via the deadenylation-dependent, deadenylation-independent, and endonucleolytic pathways, and by cytoplasmic polyadenylation, most notably indicated in early development [11]. The trans-acting cytoplasmic polyadenylation element binding protein (CPEB), which binds to the regulatory *cis*-acting sequence, the cytoplasmic polyadenylation element (CPE), in the 3’-UTR of mRNAs is implicated in the temporal regulation of translational activation and repression of mRNA in *Xenopus* and clam oocytes is [12-23]. In addition to regulating the mRNA localization of vertebrate and invertebrate maternal mRNAs, CPEB upregulation is also indicated in translational regulation occurring in hippocampal neurons, mouse synaptosomes, and the activated synapses in *Aplysia* [24-26]. In the most current model for CPEB-CPE regulation (Figure 1) in the role of translational repression (masking) and translational activation (unmasking), CPEB is continuously bound to the CPE, having a consensus sequence of U_4-5_A_1-3_U (Figure 1; image reprinted from [27] with kind permission of the authors). This consensus sequence is usually positioned at the following sites within the 3’-UTRs: 15–100 bp upstream, immediately adjacent to, or overlapping with the poly(A)^+^ signal [14,28-31]. mRNAs that are translationally repressed are referred to as dormant or “masked,” and are often characterized as having short poly(A)^+^ tails that are on the order of 20–50 nucleotides. Actively translated or “unmasked” mRNAs are characterized by long poly(A)^+^ tails of 100-150 nucleotides in length or longer [12,28]. Prior to their exit from the nucleus, mRNAs have long poly(A)^+^ tails that are shortened upon entrance into the cytoplasm by the association of CPEB, the poly(A)-specific RNase, PARN, and the poly(A)polymerase, Gld2, thereby promoting translational repression. During translational activation phosphorylation of CPEB by Aurora A results in the release of PARN and the subsequent initiation of poly(A)^+^ tail elongation by Gld2 [18,28,32,33].

Thus far, CPEB-CPE regulation of polyadenylation has not been indicated in retinal or in other adult somatic cells, except in dendrites and synapses of mice brain. CPEB-CPE regulation has also been demonstrated in dendrites of the hippocampus, in cultured neuronal synapses, and in synaptic densities in adult brains [12,24,25]. In a novel experiment to determine the extent of CPEB presence and regulation mRNAs in the brain, Wu et al. [24] compared the polyadenylation of CPE-containing α-CaMKII mRNA, which is involved in long-term potentiation of neurons during reorganization of the visual cortex when exposed to light, to that of neurofilament (NF) mRNA lacking CPE-like sequences in dark- to light-reared rats. While there was no change in the poly(A)^+^ tail lengths of the NF mRNAs, there was significant growth of the poly(A)^+^ tail lengths of the α-CaMKII mRNA in addition to increases in α-CaMKII protein after exposure to light at 30 min, 120 min, and 360 min following dark adaptation. Additional evidence of the existence of putative CPE sequences within the 3’-UTR of the octopus lens S1-crystallin mRNA and mutated optineurin (*OPTN*) genes of wide-angle glaucoma patients suggests that CPEB-CPE regulation may play a role in gene expression of some crystallins and other proteins of the eye [34,35]. Based on these data and CPEB-CPE involvement in regulation of α-CaMKII mRNA of the brain, we believe that mRNA for the cytoskeletal proteins, α-tubulin and S-crystallin, may be stored and translated when needed in the light or dark, a direct result of CPE-dependent mRNA masking and unmasking. If masking and unmasking of these mRNAs does occur, then regulatory elements or sequences should be present in their 3’-UTRs.

Here we report the presence of CPEB in dark- and light-adapted octopus retinas and the detection of a putative CPE-like sequence in the 3’-UTRs of the long S-crystallin mRNA variants isolated from the same dark- and light-adapted octopus retinas. Western blot analysis identified CPEB in retinal whole cell lysates isolated from dark- or light-adapted control octopuses at 180 min and from octopuses moved to the opposite lighting conditions at 5 min, 15 min, and 45 min timed intervals. Subsequent sequencing of 3’-UTRs of α-tubulin and of the short S-crystallin variants isolated via 3’-RACE showed no CPE or CPE-like sequences. Differences in the number of canonical poly(A)^+^ signals were also detected in the 3’-UTR of the short and long S-crystallin mRNA variants. These novel findings suggest that the differential expression of the long S-crystallin mRNA variant in octopus retinas could be mediated by CPEB-dependent polyadenylation, which, in turn, is regulated by light and dark exposure.

## Methods

### Animals

Octopuses (*Octopus bimaculoides*) were collected from the coastal waters of Southern California (Aquatic Research Consultants, San Pedro, CA) and stored in water coolers with aerated seawater. After exposure to dark and light adaptation, the animals were anesthetized on ice, their eyecups were dissected, and the lenses were removed, as recommended by the CSUDH Institutional Animal Care and Use Committee. The experimental conditions of the dark and light adaptation were as follows: Dark adaptation of 2 control octopuses for 180 min, after which the remaining 6 animals were exposed to light at 5-, 15-, and 45-min intervals; and light-adaptation of 2 control octopuses for 180 min, followed by exposure of the remaining 6 animals to dark at 5-, 15-, and 45-min intervals. Dissected eyecups were immersed in 800 µl RNA*later* (Ambion, Inc., Austin, TX) or in 800 µl 67 mM phosphate buffer, pH 7.4, and stored at -80 °C for subsequent mRNA or protein isolation.

### mRNA isolation and 3’-RACE PCR

The retinas from 16 octopuses were scraped from the sclera and the tissue homogenized in 12 volumes of Lysis Solution (Ambion, Inc., Austin, TX) with a glass Teflon homogenizer. mRNA was isolated from homogenized retinal tissue using the MicroPoly(A)Purist Kit (Ambion, Inc.). The mRNA quantity and quality were determined by UV spectrophotometry at 260 nm and 280 nm and by visualization of fluorescent smears from 0.5–12 kb on a 1% TBE agarose gel. Aliquots of 0.5 µg of mRNA were reacted with 1 µl 3’-CDS primer A, 1 µl MMLV PowerScript Reverse Transcriptase, and other reagents (5X first-strand buffer, 20 mM DTT, and 10 mM dNTP mix), as recommended in the SMART RACE cDNA Amplification Kit (Clontech, Mountain View, CA), in 10 µl reactions to generate first-strand cDNA.

#### 3’-RACE PCR

First-strand cDNA samples were amplified in 3’-RACE PCR reactions using either α-tubulin or S-crystallin gene-specific primers (GSPs), nested GSPs, and universal primer A mix from the SMART RACE cDNA Amplification Kit (Clontech).

#### Primer design

The following α-tubulin and S-crystallin GSPs were designed based on regions located 173 bp and 100 bp upstream of the TAA or TGA stop codons of α-tubulin (X15845.1 and L10110.1) and S-crystallin sequences (X74858 and M65184), respectively, of *Octopus dofleini* and *Octopus vulgaris*: α tubGSP1: 5’-GAT GTA TGC CAA GCG TGC TTT CGT TC-3’ and ScrysGSPA: 5’-ATG GGT GAC CAG ATG ACC ATG GGC-3’. The following nested GSPs (NGSPs) were designed based on regions located 76–80 bp and 100 bp upstream of the TAA stop codons of α tubulin and S-crystallin, respectively, from *O. dofleini* and *O. vulgaris*: αtubGSP2: 5’-GAG GAT TTG GCA GCT CTG GAG AAG G-3’, αtubGSP3: 5’-GCC CGT GAG GAT TTG GCA AGC TCT GGA G-3’, and ScrysGSP1: 5’-TTC TGA GCA GCT ACC CCA AAT TGC AG-3’.

#### TA-Cloning and Sequencing of 3’-RACE products

Resultant 3’-RACE PCR products were electrophoresed on l.5% low-melting agarose gels (Sigma-Aldrich, St. Louis, MO), purified using the NucleoTrap Nucleic Acid Purification Kit (Clontech), and TA-cloned into the pCR2.1-TOPO vector (Invitrogen, Carlsbad, CA). The pCR2.1-TOPO constructs were transformed into chemically competent cells, using One Shot Mach1-T1^R^, and plasmid DNA was isolated using the PureLink Quick Plasmid Miniprep Kit (Invitrogen). The presence and orientation of the 3’-RACE PCR inserts were verified by EcoRI and KpnI restriction enzyme digestion and clones positive for the presence of insert were subjected to additional PCR using M13 forward and M13 reverse primers (Invitrogen).

The quality and quantity of gel-purified PCR products were determined by UV spectrophotometry at 260 nm and 280 nm and by visualization on a 1.5% TBE agarose gel. Reactions containing 150 ng of gel-pure PCR product and 6.7 pmol of primer were brought to a total volume of 18 µl with deionized H_2_O and were sequenced in both directions, using the ABI 3730 DNA Analyzer (University of Southern California Microchemical DNA Core Facility, Los Angeles, CA).

### Post-sequence analysis of α-tubulin and S-crystallin 3’-UTR

The resultant 3’-UTR sequence data were analyzed using the Sequence Scanner v1.0 program (Applied Biosystems, Foster City, CA) to identify putative CPE sequences. The BLAST 2 sequences alignment tool (bl2seq, version 2.217) was used to confirm that isolated 3’-RACE sequences were homologous to the α-tubulin and S-crystallin mRNA sequences of *O. dofleini* and *O. vulgaris*.

### Western blot analysis

Retinal tissue from 16 octopuses were homogenized in 800 µl of 67 mM phosphate buffer, pH 7.4, and centrifuged at 18,894x g for 15 min at 4 °C. The resultant protein supernatant concentrations were quantified based on the Bradford method using the Bio-Rad Protein Assay (Bio-Rad Laboratories, Hercules, CA). Equal concentrations of total protein were diluted in 1:1 volumes of Laemmli reducing buffer (Bio-Rad Laboratories) and boiled at 100˚C for 5 min. The protein samples were loaded onto 8%, 10%, and 12% polyacrylamide gels and were subjected to SDS-PAGE for 15 min at 80 V, followed by 2-3 h at 150 V. Western blot transfer to nitrocellulose membranes occurred at 30 V overnight at 4˚C or at 100 V for 1 h at 4˚C. Blotted membranes were incubated in 20 ml of SuperBlock T20 TBS Blocking Buffer (Pierce, Rockford, IL) for 45–60 min at room temperature with gentle agitation, followed by western blot analysis using the SuperSignal West Pico Chemiluminescent Substrate Kit (Pierce). The commercially available CPEB antibody (Affinity BioReagents, Golden, CO) was used for detection at a 1:5,000 dilution in Tris Buffered Saline, pH 7.5, and 0.05% Tween-20 (TBS-T), followed by a 1:20,000 dilution of secondary horseradish peroxidase-conjugated goat antirabbit (GAR-HRP) antibody (Pierce), and visualization with the Versadoc 3000 Imager (Bio-Rad Laboratories).

#### Secondary antibody negative control blots

Blotted nitrocellulose membranes were subjected to 1:20,000 secondary GAR-HRP antibody detection alone followed by chemiluminescent visualization. PEP-171 inhibition of the CPEB antibody was performed as follows: 5 µg (5 µl) of CPEB antibody was incubated in the presence of 25 µg (50 µl) of the neutralizing peptide, PEP-171 (Affinity BioReagents), in 1 ml TBS-T at room temperature for 90 min. Following incubation, the antibody-peptide reaction was diluted in 19 ml TBS-T and used as the source for primary antibody detection of blotted nitrocellulose membranes, which were subjected to western blot analysis as described in the previous section.

#### Dephosphorylation of protein lysates

Protein samples (either 20 or 30 µg) were incubated in the presence of 1 U and 2 U, respectively, of Calf Intestinal Alkaline Phosphotase (CIP; New England Biolabs, Inc., Ipswich, MA), NEB #3 buffer (New England Biolabs, Inc.) and 1 µl 100X protease inhibitor cocktail (Cytoskeleton, Inc., Denver, CO) in 10 µl reactions at 37 °C for either 30 or 60 min intervals. Control protein samples not incubated with CIP were prepared and incubated in parallel with CIP-treated samples. Following incubation, all samples were subjected to SDS–PAGE and western blot analysis as described in the previous section.

## Results

### Analysis of α-tubulin and S-crystallin 3’-UTRs

**Figure 2:**
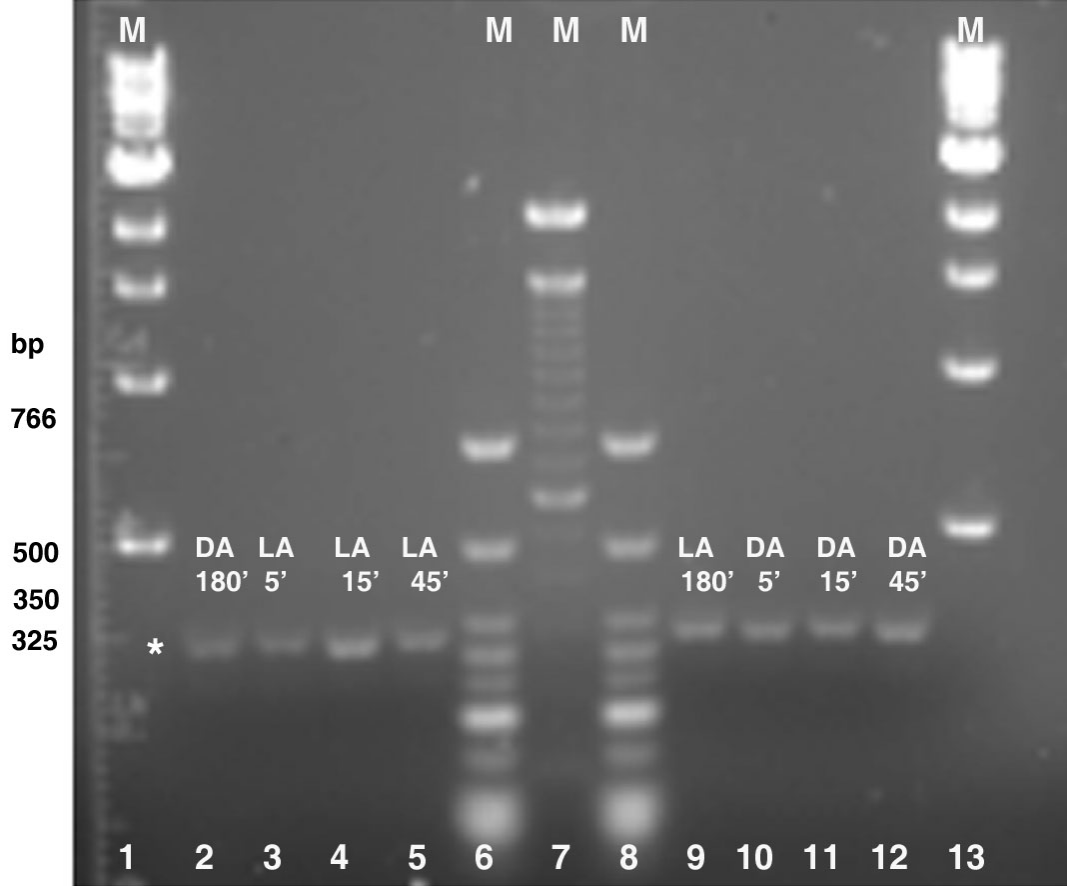
Agarose gel electrophoresis of α-tubulin 3’-RACE. Equal concentrations of mRNA used for 3’-RACE was isolated from control octopuses at 180 min of dark or light adaptation and from octopi moved to the opposite lighting conditions at 5 min, 15 min, and 45 min time intervals. Lanes 2-5 and 9-12: The asterisk indicates the approximate size of the amplified α-tubulin 3’-RACE fragment as 325 bp. The DNA marker sizes correspond to the fragments of the low molecular weight ladder (New England BioLabs Inc., Ipswich, MA) in lanes 6 and 8. Lanes 1 and 13 contain the 1 kb DNA ladder (New England BioLabs Inc.), and lane 7 contains the 100 bp DNA ladder (Invitrogen, Carlsbad, CA). The following abbreviations are used in the figure: marker (M), light-adapted (LA), and dark-adapted (DA).

**Figure 3:**
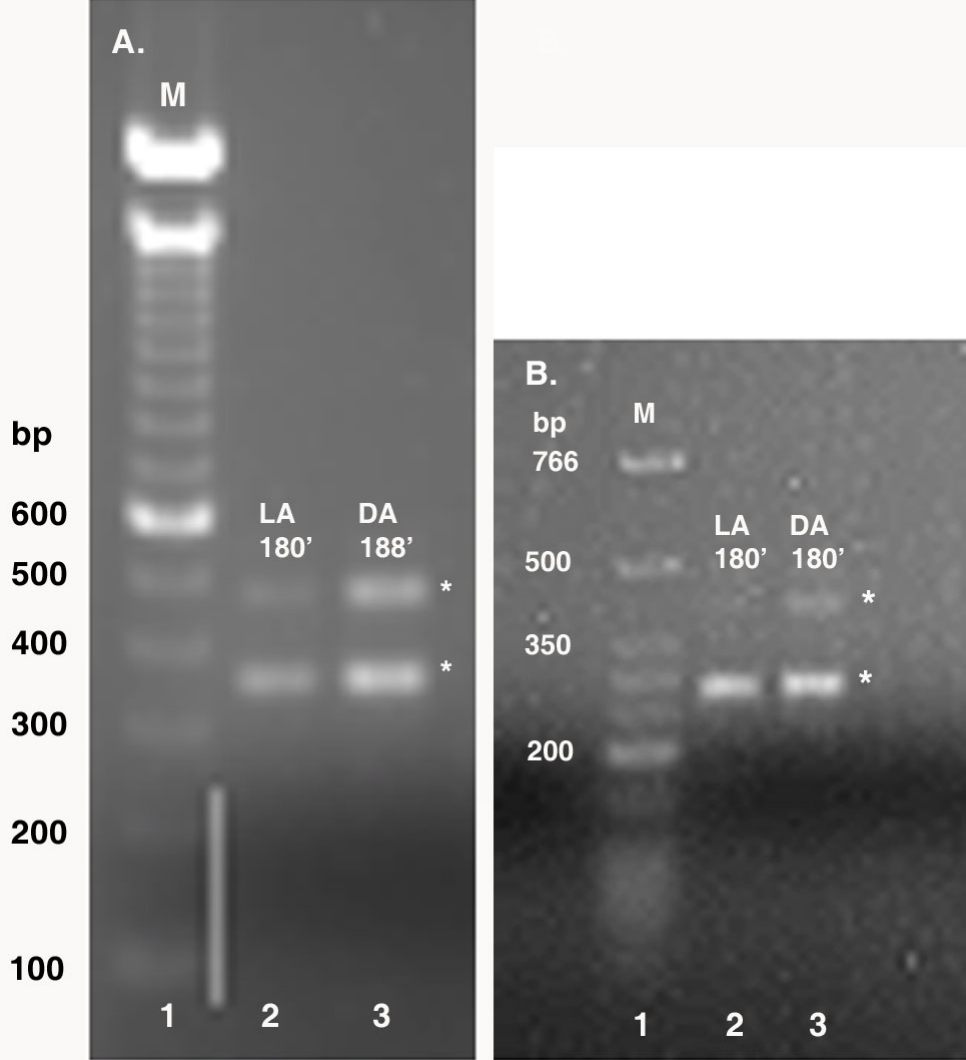
Agarose gel electrophoresis of S-crystallin 3’-RACE. Equal concentrations of mRNA used for 3’-RACE was isolated from control octopuses at 180 min of dark or light adaptation and from octopi moved to the opposite lighting conditions at 5 min, 15 min, and 45 min time intervals. Shown in this figure is the 3’-RACE for the 180 min intervals. The asterisks indicate the approximate size of the amplified S-crystallin 3’-RACE fragments. **A**: Amplification with S-crystallin GSPs generated two fragments at approximately 350 bp and 500 bp in LA and DA octopus retinas (lanes 2 and 3). **B**: Amplification with S-crystallin nested gene specific primers (NGSPs) generated two fragments at approximately 300 bp and 450 bp, and although barely visible, the 450 bp fragment is present in LA retinas (lanes 2 and 3). The DNA marker sizes (lane 1) in A and B correspond to the fragments of the 100 bp DNA and low molecular weight ladders (Invitrogen and New England BioLabs, Inc.), respectively. The following abbreviations are used in the figure: marker (M), light-adapted (LA), and dark-adapted (DA).

3’-RACE was used to identify CPE sequences in the 3’-UTRs of α-tubulin mRNA isolated from the octopuses dissected at each of the previously described experimental lighting conditions and intervals. 3’-RACE using S-crystallin primers was performed on mRNA isolated from control octopuses dark- or light-adapted for 180 min only. Amplification with the α-tubulin GSP generated α-tubulin 3’-RACE lengths of approximately 325 bp (Figure 2). Amplification with the S-crystallin GSP revealed two S-crystallin 3’-RACE lengths of approximately 350 bp (short variant) and 500 bp (long variant) in length in the 180 min dark- and light-adapted control octopus retinas (Figure 3A). S-crystallin NGSPs were used in nested 3’-RACE PCR experiments as confirmation that the multiple S-crystallin RACE fragments were real, complete products, such as splice variants or isoforms, and not artifacts resulting from non-specific priming. The persistence of multiple bands running at smaller sizes, approximately 300 bp and 450 bp, was an indication that the S-crystallin 3’-RACE PCR reactions were correctly primed (Figure 3B) and that resultant fragments were real.

The BLAST 2 sequences alignment tool (bl2seq, version 2.2.17) was used to confirm the sequence homology of isolated 3’-RACE products to α-tubulin and S-crystallin sequences of *O. vulgaris* (X15845.1, X65543.1, X65544.1, X74858.1, GenBank) and *O. dofleini* (L10110.1, M65184.1, M65185.1, M65186.1, M65187.1, GenBank). Alignment of the putative 325 bp α-tubulin fragments to the *O. dofleini* mRNA for α-tubulin revealed an overall homology of 92%. The putative 350 bp S-crystallin fragments (short variants) exhibited the following homologies: 93% homology to *O. vulgaris* mRNA for glutathione *S*-transferase, 92% homology to *O. vulgaris* mRNA for S-crystallin, and 86% homology to *O. dofleini* mRNA for lens S1-crystallin. The 500 bp S-crystallin fragments (long variants) were determined to be homologous to a wide range of known S-crystallin proteins such as *O. vulgaris* mRNA for glutathione *S*-transferase at 92%- 93% homology and *O. dofleini* lens S3-crystallin mRNA at 89%-91% homology.

​BLAST 2 sequences alignment tool (bl2seq, version 2.2.17) and Sequence Scanner v1.0 programs were also used to analyze the 3’-UTRs of isolated α-tubulin and S-crystallin mRNAs. The primers used to amplify the isolated α-tubulin and S-crystallin 3’-UTRs were positioned 173 bp and 100 bp upstream of stop codons in known *O. dofleini and O. vulgaris* α-tubulin and S-crystallin mRNAs. However, the sequence alignment of the isolated mRNAs with other known mRNAs identified the position of the α-tubulin and S-crystallin primers at 169 bp and 174 bp, respectively, upstream of the TAA or TGA stop codons in *O. bimaculoides* mRNA. Therefore, the actual lengths of the isolated 3’-UTRs were approximately 78-95 bp for α-tubulin, and 135-136 bp for short S-crystallin variants, and 190-198 bp for long S-crystallin variants.

Additional post-sequence analysis with Sequence Scanner v1.0 revealed the following: one putative hexanucleotide poly(A)^+^ signal and one poly(A)^+^ tail in the α-tubulin 3’-UTR; two putative hexanucleotide poly(A)^+^ signals and one poly(A)^+^ tail in the 3’-UTR of the short S-crystallin variants; and only one poly(A)^+^ signal and one poly(A)^+^ tail in the 3’-UTR of the long S-crystallin variants (Table 1).

**Table 1:** Summary of the α-tubulin and S-crystallin 3’-UTR analysis.

**Isolated 3’-UTRs**	**Poly(A)^+^ signal**	**Poly(A) tail**	**Non-canonical Poly(A)^+^ signal**	**CPE or CPE-like sequences**	**U- or UA-rich**
α-tubulin	1	1	0	0	No
S-crystallin, short variant	2	1	0	0	No
S-crystallin, long variant	1	1	0	UUUAACA	No

The data presented enumerate some common 3'-UTR regulatory and CPE-like sequences found within isolated α-tubulin and S-crystallin mRNAs.

While CPE or CPE-like sequences were not detected in the 3’-UTRs of either α-tubulin or of the short S-crystallin variants, the following CPE-like sequence was detected in the 3’-UTRs of the long S-crystallin variants: UUUAACA (Table 1). The short and long S-crystallin 3’-RACE variants were also subjected to a BLAST alignment to determine if there were sequence variations that were responsible for the difference in the numbers of poly(A)^+^ signal existing within their 3’-UTRs. The sequence alignments between the short and long S-crystallin 3’-RACE variants showed that homology was relatively conserved in the sequence preceding the stop codons and immediately following the poly(A)^+^ signal. In short and long S-crystallin 3’-RACE variants isolated from light-adapted retinas, the following homologies existed: 85% in regions upstream of the stop codon; 0% in the non-conserved regions; and 86% in regions downstream of the poly(A) signal. In short and long S-crystallin 3’-RACE variants isolated from dark-adapted retinas, the following homologies existed: 82% in regions upstream of the stop codon; 0% in the non-conserved regions; and 85% in regions downstream of the poly(A) signal.

### Identification of CPEB in dark- and light-adapted octopus retinas

**Figure 4:**
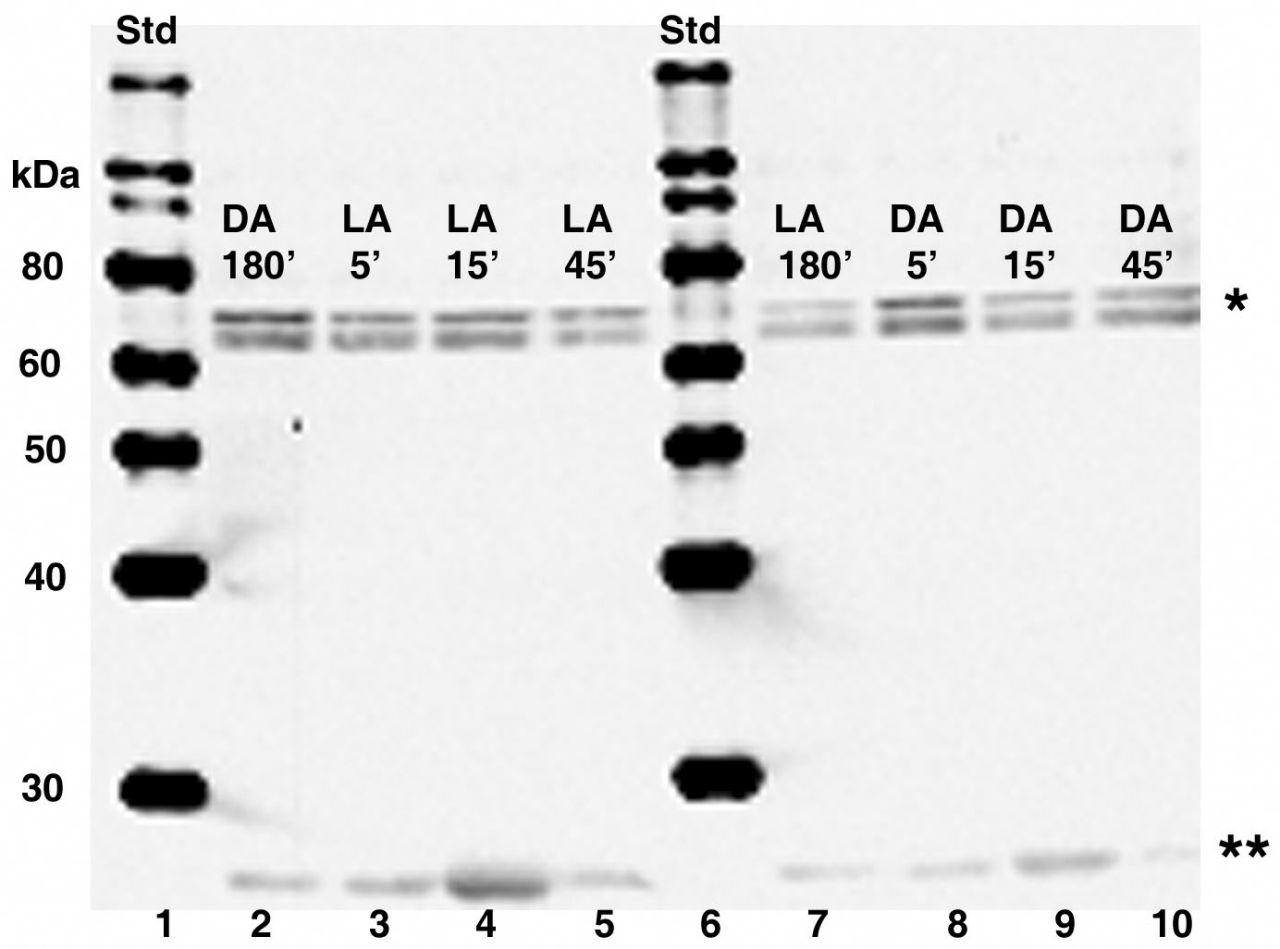
CPEB protein is detected in dark-adapted and light-adapted octopus retinas. Equal concentrations of total retinal protein were isolated from control octopi at 180 min of dark or light adaptation and from octopuses moved to the opposite lighting conditions at 5 min, 15 min, and 45 min time intervals. Lanes 2-5 and 7-10: Two major immunoreactive bands are present between 60-80 kDa (single asterisk), while a smaller one is detected below 30 kDa (double asterisk). The molecular weight sizes correspond to the fragments of the Magic Mark protein XP standard (Invitrogen) in lanes 1 and 6. The following abbreviations are used in the figure: standard (Std), light-adapted (LA), and dark-adapted (DA).

Equal concentrations of total retinal protein isolated from dark- or light- adapted control *Octopus bimaculoides* at 180 min and from animals moved to the opposite lighting conditions at 5 min, 15 min, and 45 min intervals were subjected to SDS–PAGE and blotted to nitrocellulose membranes. The resulting immunoblots were subjected to detection with the CPEB antibody, which recognized the following human and recombinant mouse sequence at 62 kDa: C-H(545) S-M-E-G-L-R-H-H-S-P-L-M-R-N-Q-K-N(562). The CPEB antibody recognized two single immunoreactive bands between 60–80 kDa, with the smaller of the two bands sometimes migrating as doublet. A third immunoreactive band also migrated below 30 kDa (Figure 4), which may be attributable to degradation of the putative higher molecular weight CPEB proteins rather than to nonspecific binding. With the exception of the third immunoreactive band, the intensity of putative higher molecular weight CPEB protein bands appeared constant at each time point, which supports the current hypothesis that CPEB is always present and bound to the CPE consensus sequence [14,28-31]. Since demonstration of constant or differential expression of CPEB was not the intent of this paper, use of β-actin as a control for loading was not performed. However previous results have shown β-actin mRNA and protein to be constant at all time points during dark and light adaptation (data not shown).

**Figure 5:**
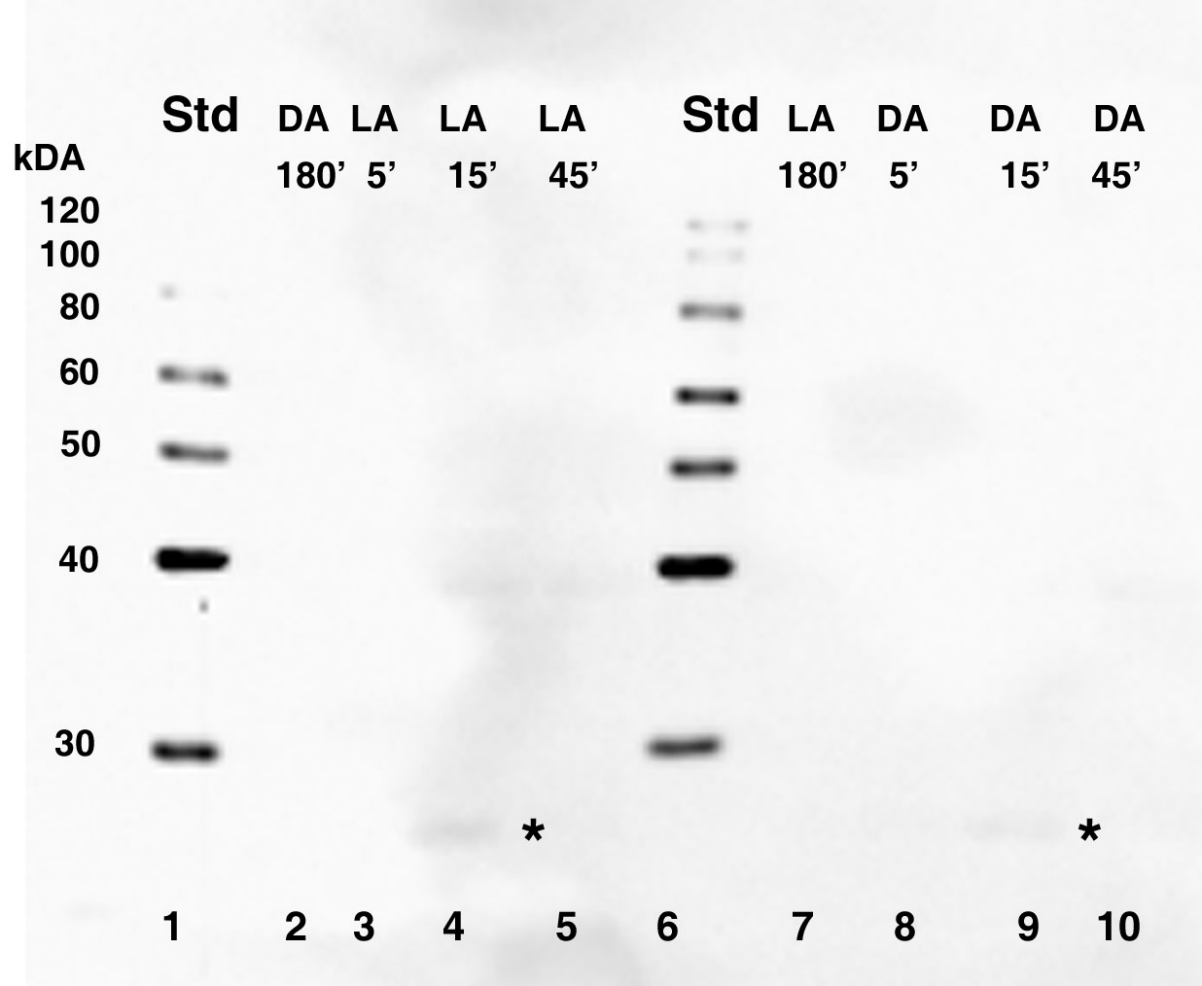
Western blot showing secondary GAR-HRP antibody detection only. The detection of CPEB in dark-adapted (DA) and light-adapted (LA) retinas does not result from nonspecific antibody binding. Equal concentrations of total retinal protein was isolated from control octopi at 180 min of dark or light adaptation and from octopi moved to the opposite lighting conditions at 5 min, 15 min, and 45 min time intervals. Lanes 2-5 and 7-10: The signals of the two major immunoreactive bands present between 60-80 kDa were not detected. Also slightly visible is a third smaller signal migrating below 30 kDa (asterisk). The molecular weight sizes correspond to the fragments of the Magic Mark XP protein standard (Invitrogen) in lanes 1 and 6. In the figure, standard is abbreviated Std.

**Figure 6:**
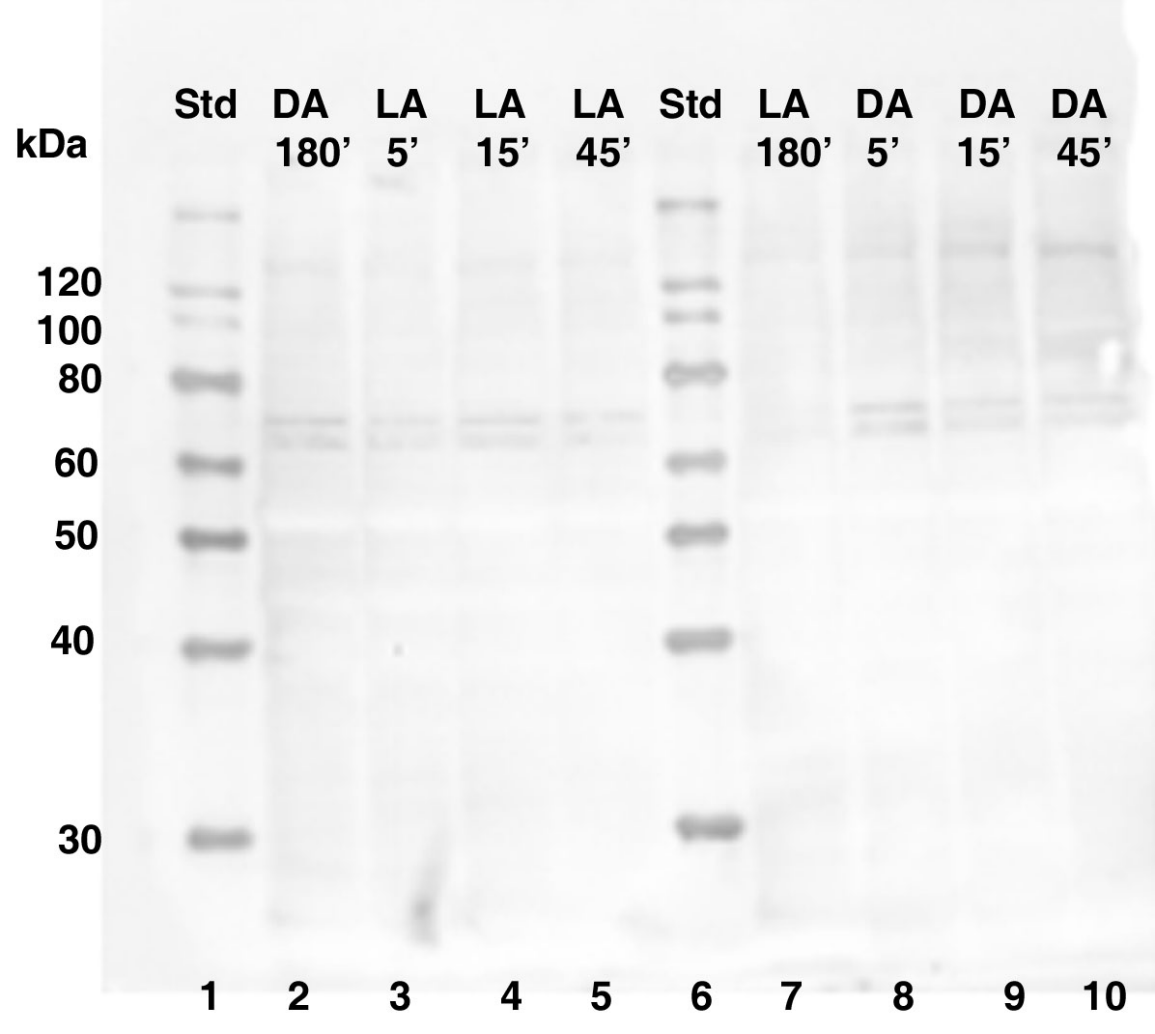
Western blot showing the effect of inhibition of the CPEB antibody with PEP-171 neutralizing peptide. Equal concentrations of total retinal protein was isolated from control octopi at 180 min of dark or light adaptation and from octopi moved to the opposite lighting conditions at 5 min, 15 min, and 45 min time intervals. Lanes 2-5 and 7-10: The signal of the two major immunoreactive bands present between 60-80 kDa is remarkably reduced, as is the signal of smaller band is detected below 30 kDa. The molecular weight sizes correspond to the fragments of the Magic Mark XP protein standard (Invitrogen) in lanes 1 and 6. The following abbreviations are used in the figure: standard (Std), light-adapted (LA), and dark-adapted (DA).

To rule out the possibility of nonspecific primary antibody binding, negative control blots were incubated in the presence of secondary GAR-HRP antibody only. Secondary GAR-HRP antibody detection alone resulted in the complete absence of the two putative immunoreactive bands migrating between 60-80 kDa (Figure 5). Also barely visible were the immunoreactive bands running below 30 kDa. Since a CPEB control protein was not commercially available for use as a positive loading control, PEP-171 neutralization of the CPEB antibody was used to further confirm that non-specific primary antibody binding was not significant. When CPEB antibody is competitively inhibited by PEP-171 neutralizing peptide for 1 h at room temperature, the positive signal is significantly reduced (Figure 6). As expected with any type of inhibition assay, the main positive signal doesn’t always disappear completely. Therefore, the main bands migrating between 60 and 80 kDa were interpreted as the putative target protein, CPEB, since there was a significant decrease in their signals.

**Figure 7:**
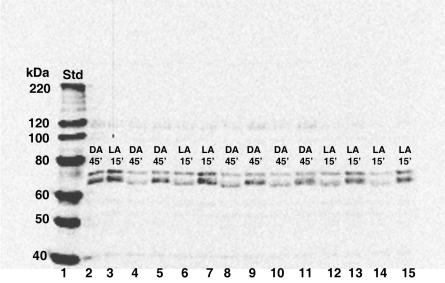
Western blot showing the effect of CIP on CPEB size. Equal concentrations of total retinal protein isolated from a single 15 min light-adapted (LA15) retinal sample and a single 45 min dark- adapted (DA45) retinal sample were chosen at random. Lanes 2-3: The signals of the two major immunoreactive bands migrating between 60-80 kDa are detected in the DA45 and LA15 lacking CIP and 100x protease inhibitors. Lanes 4-15: All samples in lanes 4-15 were treated in the presence of 100X protease inhibitors. DA45 and LA15 samples in lanes 4 and 6 were incubated in the presence of CIP (0.1 μl) for 30 min. Lanes 5 and 7 contain DA 45 and LA15 samples incubated in the absence of CIP for 30 min. DA45 and LA15 samples in lanes 8, 10, 12, and 14 were incubated in the present of CIP for 1 h. Samples in lanes 8 and 12 contain 0.2 μl of CIP and samples in lanes 10 and 14 contain 0.1 μl of CIP. Lanes 9, 11, 13, and 15 contain DA 45 and LA15 samples incubated in the absence of CIP for 1 h. The molecular weight of the smaller of the two immunoreactive bands migrating between 60-80 kDa is remarkably reduced in all samples incubated in the presence of 0.1 μl or 0.2 μl of CIP. Although not visible in this 12% blot, the size of the 30 kDa band is not affected. The molecular weight sizes (lane 1) correspond to the fragments of the Magic Mark XP protein standard (Invitrogen). The following abbreviations are used in the figure: standard (Std), light-adapted (LA), and dark-adapted (DA).

Protein samples were incubated in the presence of alkaline phosphotase, calf intestinal (CIP) to determine if the migration pattern of the two main signals between 60-80 kDa was a result of phosphorylation. While there appeared to be no change in the molecular weight of the top immunoreactive band, the molecular weight of the lower immunoreactive band was visibly decreased (Figure 7).

## Discussion

Coincidental with the observed morphological changes of the dark- and light-adapted rhabdomeres are dynamic differences in α-tubulin and S-crystallin expression in the dark-and light-adapted octopus retinas. In enlarged dark-adapted rhabdomeres, increases of α-tubulin and S-crystallin protein are paralleled by decreases of these mRNAs. Meanwhile, in shrunken light-adapted rhabdomeres, decreases of α-tubulin and S-crystallin protein are paralleled by increases of these mRNAs [1-4]. While previous studies have also reported the differential expression of tubulins and crystallins in retinas, there are no concrete explanations elucidating the mechanisms or regulation by which this expression occurs [5-7]. Evidence of CPE-dependent cytoplasmic polyadenylation in maternal mRNAs of *Xenopus* oocytes and embryos and in the α-CaMKII mRNA in the hippocampal region of the brain of light-exposed rats led us to question the existence of the same type of post-transcriptional regulation of α-tubulin and S-crystallin in light- and dark-adapted octopus retinas. Further fueling our inquiry that CPE-dependent cytoplasmic polyadenylation of retinal mRNAs could occur is the presence of a putative CPE in the 3’-UTR of the octopus lens S1-crystallin mRNA [35]. Also, more recent evidence has shown that mutations resulting from a certain polymorphism in the OPTN gene of wide-angle glaucoma patients resulted in the creation of putative CPE binding sites [34].

We have identified two putative CPEB immunoreactive bands, the lower of which undergoes phosphorylation, in light- and dark-adapted octopus retinas (Figure 4 and Figure 7). An additional band migrating below 30 kDa could be interpreted as either cleavage products or splice variant forms of the two larger putative CPEB bands. 3’-RACE revealed the following: α-tubulin fragments of approximately 325 bp (Figure 2) with 3’-UTR lengths of approximately 78–95 bp; S-crystallin 3’-RACE fragments of approximately 350 bp (short variant) with 3’-UTR lengths of approximately 135-136 bp; and a S-crystallin 3’-RACE fragments of approximately 500 bp (long variant) with 3’-UTR lengths of approximately 190-198 bp. Since octopus and squid S-crystallins are known to be homologous to glutathione *S*-transferase (GST) and possess some inherent GST activity, it is likely that the S-crystallin 3’-RACE PCR fragments are isoforms of the S-crystallin multigene family [36]. These findings, in addition to the existence of the CPE-like sequence, UUUAACA in the 3’-UTR of the long S-crystallin mRNA variant (Table 1), suggest that cytoplasmic polyadenylation may have a role in the differential expression of some S-crystallin isoforms or of other mRNAs in the octopus retina. CPE or CPE-like binding sites were absent from the 3’-UTR of α-tubulin and of the short S-crystallin variants, indicating that other forms of post-transcriptional regulation may be involved in their differential expression (Table 1).

Additional variations in number or in the absence or presence of other regulatory sequences in the 3’-UTR may account for the differential expression of α-tubulin and S-crystallin. Two poly(A)^+^ signals were detected in the 3’-UTR of the short S-crystallin variant, and only one poly(A)^+^ signal was detected in the 3’-UTRs of α-tubulin and of the long S-crystallin variant (Table 1). It is suggested that mRNAs containing one or more non-canonical variant poly(A)^+^ signals are translated at rates much lower than those mRNAs containing the canonical AAUAAA poly(A)^+^ signal, thus linking poly(A)^+^ signal variation to differential gene expression. Differences in the quantity and sequence permutations of poly(A)^+^ signals are also known to contribute to the downregulation or upregulation of mRNA, as in the case of the laminin-A chain, which possesses the usual, but non-canonical AUUAAA hexanucleotide sequence [37-39]. Beaudoing et al. compared 8,775 human 3’-UTRs to 157,775 expressed sequence tags and found AAUAAA (58.2% ) and AUUAAA (14.9%) to be the most frequent poly(A)^+^ signals in 4,344 UTRs; 26.8% consisted of less observed, non-canonical poly(A)^+^ signals [38]. Moreover, 26.8% of the 3’-UTRs contained two or more non-canonical polyadenylation signals. While we did not perform any statistical quantitative analyses of the S-crystallin 3’-UTRs lengths, it was apparent that the 500 bp (long variant) 3’-RACE S-crystallin isoform was much less abundant than the 350 bp (short variant) 3’-RACE isoform (Figure 3). The more abundant 350 bp 3’-RACE S-crystallin isoform also contained more than one canonical polyadenylation signal in its 3’-UTR, suggesting that the alternative use of either canonical poly(A)^+^ signal may be responsible for differential expression of S-crystallin.

Other known post-transcriptional regulators of differential gene expression are micro RNAs (miRNAs) and short interfering RNAs (siRNAs). Both miRNAs and siRNAs are small RNAs ranging 21 to 25 and 21 to 23 nucleotides in length, respectively, and are hypothesized to repress translation by binding to complementary sequences with 3’-UTR regions. While previous research has suggested that the miRNAs function by interacting with the actively translating mRNAs in polyribosome complexes without affecting mRNA stability, other research suggests that miRNAs regulate gene expression through mRNA degradation [40-42]. The same dynamic miRNA regulation of the *lin-41*, *lin-14*, and *lin-28* mRNAs could explain the differential gene patterns α-tubulin and S-crystallin mRNAs in the octopus retinas, especially those lacking CPE and CPE-like sequences. Higher levels of α-tubulin and S-crystallin mRNA correspond with lower levels of the protein in light-adapted retinas, which is indicative of regulation by miRNAs as effectors of translational repression. However, lower levels of α-tubulin and S-crystallin mRNA paralleling the higher levels of protein in dark-adapted retinas would suggest that miRNAs function as effectors of mRNA degradation.

The detection of CPEB in dark- and light-adapted octopus retinas and the identification of a CPE-like sequence existing within the 3’-UTR of the long S-crystallin mRNA variants suggest that CPE-dependent cytoplasmic polyadenylation may be involved in the activation of masked S-crystallin mRNA. CPEB-mediated control of differential α-tubulin expression is unlikely since the isolated α-tubulin 3’-UTRs lacked putative CPE or CPE-like sequences. Further research is also needed to address the functional significance of the CPE-like sequence existing within the 3’-UTR of the long S-crystallin mRNA variant and whether CPEB can interact with this CPE-like sequence. Equal attention should be directed toward deciphering the significance of the multiple poly(A)^+^ signals existing within the 3’-UTR of the short S-crystallin mRNA variant and of the single poly(A)^+^ signal existing within the 3’-UTR of the long S-crystallin mRNA variant. Polyadenylation (PAT) assays would further determine if the increases of α-tubulin and S-crystallin protein observed in the dark result from changes in the poly(A)^+^ tail length or synthesis of α-tubulin and S-crystallin mRNA. Finally, an investigation of the possible roles of miRNAs and siRNAs as translational inhibitors or effectors of α-tubulin and S-crystallin mRNA degradation in LA retinas would provide further insight as to the post-transcriptional or translational regulation of not only these two mRNAs, but of other known and novel mRNAs of the octopus retina.
